# Implementation of evidence-based weekend service recommendations for allied health managers: a cluster randomised controlled trial protocol

**DOI:** 10.1186/s13012-018-0752-7

**Published:** 2018-04-24

**Authors:** Mitchell N. Sarkies, Jennifer White, Meg E. Morris, Nicholas F. Taylor, Cylie Williams, Lisa O’Brien, Jenny Martin, Anne Bardoel, Anne E. Holland, Leeanne Carey, Elizabeth H. Skinner, Kelly-Ann Bowles, Kellie Grant, Kathleen Philip, Terry P. Haines

**Affiliations:** 1 Monash University and Monash Health Allied Health Research Unit, Kingston Centre, 400 Warrigal Road, Heatherton, Victoria 3192 Australia; 2 Monash University and Monash Health Allied Health Research Unit, Kingston Centre, 400 Warrigal Road, Heatherton, Victoria 3202 Australia; 3 La Trobe Centre for Sport and Exercise Medicine Research, La Trobe University, Bundoora, 3086 Australia; 4 North Eastern Rehabilitation Centre, Healthscope Australia, Melbourne, Australia; 5 Allied Health Clinical Research Office, Eastern Health, Box Hill, 3128 Australia; 6 Peninsula Health, 4 Hastings Rd, Frankston, Victoria 3199 Australia; 7 Department of Occupational Therapy, Monash University, Building G, McMahons Road, Frankston, Victoria 3199 Australia; 8 School of Arts, Social Sciences and Humanities, Swinburne University, Hawthorn Campus, John St, Hawthorn, Victoria 3122 Australia; 9 Department of Management and Marketing, Swinburne University, BA 1224 Hawthorn Campus, John St, Hawthorn, Victoria 3122 Australia; 10 Alfred Health and La Trobe University, 99 Commercial Rd, Melbourne, 3004 Australia; 11 Occupational Therapy, School of Allied Health, La Trobe University, Bundoora, Victoria 3086 Australia; 12 Florey Institute of Neuroscience and Mental Health, Neurorehabilitation and Recovery, Melbourne Brain Centre, 245 Burgundy Street, Heidelberg, Victoria 3084 Australia; 13 Department of Health and Human Services, Melbourne, Victoria Australia; 14 Monash University, Level 3, Building G, Peninsula Campus, McMahons Rd, Frankston, Victoria 3199 Australia

**Keywords:** Implementation, Research, Evidence, Allied health, Weekend, Decision-making, Evidence-informed decision-making, Resource allocation, Knowledge broker, Recommendation

## Abstract

**Background:**

It is widely acknowledged that health policy and practice do not always reflect current research evidence. Whether knowledge transfer from research to practice is more successful when specific implementation approaches are used remains unclear. A model to assist engagement of allied health managers and clinicians with research implementation could involve disseminating evidence-based policy recommendations, along with the use of knowledge brokers. We developed such a model to aid decision-making for the provision of weekend allied health services. This protocol outlines the design and methods for a multi-centre cluster randomised controlled trial to evaluate the success of research implementation strategies to promote evidence-informed weekend allied health resource allocation decisions, especially in hospital managers.

**Methods:**

This multi-centre study will be a three-group parallel cluster randomised controlled trial. Allied health managers from Australian and New Zealand hospitals will be randomised to receive either (1) an evidence-based policy recommendation document to guide weekend allied health resource allocation decisions, (2) the same policy recommendation document with support from a knowledge broker to help implement weekend allied health policy recommendations, or (3) a usual practice control group. The primary outcome will be alignment of weekend allied health service provision with policy recommendations. This will be measured by the number of allied health service events (occasions of service) occurring on weekends as a proportion of total allied health service events for the relevant hospital wards at baseline and 12-month follow-up.

**Discussion:**

Evidence-based policy recommendation documents communicate key research findings in an accessible format. This comparatively low-cost research implementation strategy could be combined with using a knowledge broker to work collaboratively with decision-makers to promote knowledge transfer. The results will assist managers to make decisions on resource allocation, based on evidence. More generally, the findings will inform the development of an allied health model for translating research into practice.

**Trial registration:**

This trial is registered with the Australian New Zealand Clinical Trials Registry (ANZCTR) (ACTRN12618000029291). Universal Trial Number (UTN): U1111-1205-2621.

**Electronic supplementary material:**

The online version of this article (10.1186/s13012-018-0752-7) contains supplementary material, which is available to authorized users.

## Background

### Background and rationale

One of the challenges of evidence-based healthcare worldwide is to effectively and efficiently translate the findings of research into practice. Patient outcomes, patient satisfaction, cost-effectiveness, and quality outcomes benefit from evidence-informed decision-making [1–3]. Local healthcare policies that foster the timely translation of research findings to behaviour change can facilitate evidence-based practice [4–7]. In some cases, allied health policy and practice do not directly reflect current research evidence [8–12]. The delay in the translation of research into practice has also been documented for the medical [13] and nursing [14] professions, where it can take over 10 years for new scientific discoveries to enter day to day clinical practice [15, 16].

Allied health professionals generally have positive attitudes towards evidence-informed decision-making [8–10, 17–19]. Research receptivity and capability among allied health professionals are also influenced by organisational characteristics such as team dynamics, a culture of acceptance or resistance to change, and managerial support [20–22]. Allied health policy-makers and managers can influence these organisational factors and facilitate the translation of research into policy and practice [23]. However, they do not always have the training or access to knowledge transfer resources to assist them to engage effectively with research implementation [24, 25].

Implementation research has sought to develop strategies to reduce the gap between scientific evidence and practice [26]. A recently published systematic review identified 32 studies examining a number of different research implementation strategies for allied health professionals [27]. Education as a single strategy was most frequently evaluated, yet was not always successful in facilitating desired behaviour change [27]. Isolated educational strategies targeting individual professionals may not always meet the needs of complex organisational structures and multiple levels of decision-making involved in adopting an innovation. Providing resources to assist evidence-informed healthcare policy and management decisions may also facilitate behaviour change [28]. Slade et al. [3] highlighted the importance of allied health managers in fostering a research culture to embed evidence-based practice.

Developing evidence-based policy recommendations for allied health decision-makers has the potential to increase engagement with research implementation [29]. Short documents, which communicate key research findings in an accessible format, are one of the few research implementation strategies evaluated for use by resource allocation decision-makers, such as allied health managers [3, 23, 30, 31]. Single research implementation strategies have been reported as less successful than multifaceted approaches in some settings [32].

More interactive strategies may complement the provision of evidence-based policy recommendations, particularly in health services without a strong research culture [33]. One such interactive strategy is the use of knowledge brokers to work collaboratively with stakeholders, promoting the transfer and exchange of information [34]. Indeed, in Canada, many public health organisations have adopted knowledge broker roles as linking agents and capacity builders [35]. This is despite limited evidence to support their benefits [36]. Further high quality empirical research is needed to evaluate this particular implementation resource that could be provided to allied health policy-makers and managers to support the translation of research into practice.

### Implementation context

One area of allied health policy and practice that could better align with the current research evidence is the provision of allied health services to hospital wards during weekends. Routinely throughout the world, allied health services including physiotherapy, speech and language therapy, occupational therapy, social work, nutrition and dietetics, and podiatry, are delivered Monday to Friday. In some parts of the world, allied health services are also provided on Saturdays and Sundays [37–40]. Saturday physiotherapy services are the most common form of allied health provided outside business hours internationally [39, 41]. Only 30% of sub-acute hospitals provide weekend physiotherapy, despite evidence suggesting the provision of after-hours or weekend rehabilitation improves outcomes in the sub-acute rehabilitation setting [41–43]. Research implementation strategies could inform weekend allied health resource allocation decisions to better align policy and practice with contemporary research evidence.

### Objectives

This protocol outlines the design and methods for a multi-centre cluster randomised controlled trial to evaluate the success of select research implementation strategies for promoting evidence-informed weekend allied health resource allocation decisions by hospital managers. The implementation strategies will guide allied health managers in deciding how resources for provision of allied health services on weekends can be allocated between general medical and surgical, and sub-acute rehabilitation wards.

The resource allocation decision will be based on the following question: “How should resources for the provision of allied health services on weekends be allocated between general acute medical/surgical and sub-acute rehabilitation wards?”

## Methods

### Trial design

This multi-centre study will be evaluated using a three-group matched (based on health service regional status) parallel cluster randomised controlled trial. A three-group design will allow the comparison of two different research implementation strategies with a control. Stratification will be based on self-reported health service geographical classification as metropolitan or regional (including rural and remote), and clustering will occur at the level of weekend allied health resource allocation decision-making within each health service (e.g. health service level or hospital level).

This evaluation will be based on the Kirkpatrick Evaluation Model Hierarchy framework, which has four outcome levels that are designed as a sequence of ways to evaluate training programs [44, 45]. This study will focus on behaviour change outcomes in the third category.

### Study setting

The study sample will be drawn from Australian and New Zealand hospitals. Public or private, acute and sub-acute hospitals providing inpatient allied health services will be eligible for inclusion, with a representation of hospitals from both metropolitan and regional geographic classifications sought. Specialist hospitals such as maternity hospitals, paediatric hospitals, cancer centres, mental health and palliative care hospitals will be excluded. These hospitals will be excluded as no research regarding weekend allied health provision has been identified in these settings.

### Eligibility criteria

Allied health managers responsible for weekend allied health resource allocation decisions will be eligible for inclusion. All allied health professions currently providing an inpatient service to acute general medical and surgical wards, and sub-acute rehabilitation wards are eligible. A representation of the different allied health professions (e.g. physiotherapy, occupational therapy, speech pathology, dietetics, podiatry, psychology, exercise physiology, and social work) will be sought. We shall include those who currently provide weekend allied health services as well as those who do not currently provide services, but could potentially introduce these services.

### Interventions

Three intervention groups will be compared: control strategy group, implementation strategy group 1, and implementation strategy group 2. A summary of the intervention conditions described according to the Template for Intervention Description and Replication (TIDieR) guidelines is provided in Table 1 [46]. The two research implementation strategies were designed according to factors perceived to be associated with effective strategies and the inter-relationship between these factors to establish an imperative for change, build trust, develop a shared vision, and action a change mechanism, supported by effective employment of communication strategies and provision of resources to support change [23].

**Table 1:** Intervention conditions according to the TIDieR guidelines

TIDieR criteria	Control group	Implementation strategy group 1	Implementation strategy group 2
Item 1. “Brief name: provide the name of a phrase that describes the intervention”	Usual practice control group	Evidence-based policy recommendation document	Evidence-based policy recommendation document and a knowledge broker
Item 2. “Why: describe any rationale, theory, or goal of the elements essential to the intervention”	Usual practice is the model of weekend allied health resource allocation decision-making at the research location. This serves as a pragmatic reference standard for implementation research	The evidence-based policy recommendation document will communicate research findings in an accessible format to facilitate evidence informed decision making [43]. This will be achieved by embedding an understanding of the political context within design, providing quality evidence communicated through a credible messenger, and fostering active engagement and linkages between policy-makers and researchers [44].	The evidence-based policy recommendation document will be the same as that provided to implementation strategy group 1. In addition, the knowledge broker will act as an intermediary agent to facilitate the transfer and exchange of relevant information between researchers and healthcare decision-makers to promote evidence informed decision-making [48, 49]. The knowledge broker will undertake activities focused on identifying and engaging with decision-makers, facilitating collaboration, identifying and obtaining relevant information, facilitating development of analytic and interpretive skills, creating research implementation resources, project coordination, communication and information sharing, network development, evaluating change, and supporting sustainability [48].
Item 3. “What (materials): Describe any physical or informational materials used in the intervention, including those provided to participants or used in intervention delivery or in training of intervention providers. Provide information on where the materials can be accessed (e.g. online appendix, URL).”	There will be no materials provided to the control group during the study period. Participants will be able to use materials ordinarily available for resource allocation decisions at their discretion.	The evidence-based policy recommendation document provided will be constructed in a simple 1:3:25 format developed by the Canadian Health Services Research Foundation [55]. It allows for a one-page outline of key messages, a three-page executive summary, and 25 pages presenting the report findings and methodology.	Participants will be provided with the same evidence-based policy recommendation document as implementation strategy group 1. Participants in implementation strategy group 2 will also be provided with access to a knowledge broker who may deliver educational materials including plain English summaries, slides, and handouts. Scientific abstracts and full-text journal articles relevant to the weekend allied health resource allocation decision may also be provided as applicable.
Item 4. “What (procedures): Describe each of the procedures, activities, and/or processes used in the intervention, including any enabling or support activities.”	Weekend allied health resource allocation decisions will follow usual practice conditions according to pre-existing individual and organisational processes.	The evidence-based policy recommendation document will be emailed to participants after random group allocation. This document was developed by project investigators through a consensus building approach and reviewed by a key stakeholder committee comprised of health professionals, managers, consumers, carer representatives, policy-makers, and academics.	The same version of the evidence-based policy recommendation document provided to implementation strategy group 1 will be emailed to participants after random group allocation. The knowledge broker will offer an initial consultation to perform an individual, organisational, and external environment (e.g. government policy) needs assessment, and develop a 12-month plan. One webinar session will be offered within the first 6 months depending on allied health manager availability, and monthly follow-up contact will also be offered.
Item 5. “Who: For each category of intervention provider (e.g. psychologist, nursing assistant), describe their expertise, background and any specific training given.”	Participants may consult a variety of individuals at their discretion.	A team of tertiary qualified academics, clinicians, and policy-makers from healthcare and business management backgrounds in Victoria, Australia produced and endorsed the evidence-based policy recommendation document.	A team of tertiary qualified academics, clinicians, and policy-makers from healthcare and business management backgrounds in Victoria, Australia produced and endorsed the evidence-based policy recommendation document. In addition, one knowledge broker with a PhD level qualification, from an allied health professional background, with research experience, currently employed as a post-doctoral research fellow will be recruited for this implementation strategy.
Item 6. “How: Describe the modes of delivery (e.g. face-to-face or by some other mechanism, such as internet or telephone) of the intervention and whether it was provided individually or in a group.”	Usual practice conditions may involve participants accessing information via internet, telephone, or face to face when making resource allocation decisions.	An electronic evidence-based policy recommendation document will be provided via email.	An electronic evidence-based policy recommendation document will be provided via email. The 1:1 initial knowledge broker consultation will be offered via telephone, videoconference, or face to face (where available) as per participant preference. The group-based webinar session will be offered via video or audio and follow-up contact will be offered via email or telephone (as per participant preference).
Item 7. “Where: Describe the type(s) of location(s) where the intervention occurred, including any necessary infrastructure or relevant features.”	Usual practice conditions are likely to involve participants making decisions at their place of work.	An electronic version of the evidence-based policy recommendation document will be delivered via email. Therefore, participants may be able to access at the location of their choice. This is most likely to be accessed at their place of work, in an acute or sub-acute hospital.	An electronic version of the evidence-based policy recommendation document will be delivered via email. Therefore, participants may be able to access at the location of their choice. This is most likely to be accessed at their place of work, in an acute or sub-acute hospital. The knowledge broker contact will occur via webinar, telephone, or email. Therefore, participants may be able to access at the location of their choice. This is most likely to be accessed at their place of work in an acute or sub-acute hospital. If the initial consultation can be arranged face to face, this will occur at a location convenient to both the participant and the knowledge broker, most likely at the participant’s place of work.
Item 8. “When and How Much: Describe the number of times the intervention was delivered and over what period of time including the number of sessions, their schedule, and their duration, intensity or dose.”	12-month wait list of usual practice conditions. The evidence-based policy recommendation document will be provided upon study completion.	One evidence based policy recommendation document will be provided to participants after random group allocation for the duration for the 12-month intervention period.	One evidence-based policy recommendation document will be provided to participants after random group allocation for the duration for the 12-month intervention period. The knowledge broker will provide one 60-min initial consultation, one 60-min group webinar, and one follow-up contact each month for the 12-month intervention period.
Item 9. “Tailoring: If the intervention was planned to be personalised, titrated or adapted, then describe what, why, when, and how.”	Usual practice conditions allow participants to take various approaches when making resource allocation decisions. These can be altered at participant discretion as per organisation policy and practice.	There is no adaptation planned for the evidence-based policy recommendation document during the study period.	There is no adaptation planned for the evidence-based policy recommendation document during the study period. The knowledge broker role is iterative in nature. Interaction will be tailored to the needs of the participants at the discretion of the knowledge broker based on their professional judgement.
Item 10. “Modifications: If the intervention was modified during the course of the study, describe the changes (what, why, when, and how).”	Not applicable for protocol	Not applicable for protocol	Not applicable for protocol
Item 11. “How Well (planned): If intervention adherence or fidelity was assessed, describe how and by whom, and if any strategies were used to maintain or improve fidelity, describe them.”	Adherence or fidelity will not be assessed in the usual practice control group, as no implementation strategy will be provided during the study period.	Whether or not participants read the evidence-based policy recommendation document will be explored in the 12-month follow-up qualitative interviews.	Whether or not participants read the evidence-based policy recommendation document will be explored in the 12-month follow-up qualitative interviews. Adherence to the knowledge broker component of the implementation strategy group 2 intervention will be monitored via the knowledge broker diary kept for the 12-month period.
Item 12. “How Well (actual): If intervention adherence or fidelity was assessed, describe the extent to which the intervention was delivered as planned.”	Not applicable for protocol	Not applicable for protocol	Not applicable for protocol

#### Control strategy group

The control strategy group will involve a 12-month wait-list for the provision of an evidence-based policy recommendation document at trial completion. This group will involve usual practice conditions, as per each health services usual decision-making process.

#### Implementation strategy group 1: provision of an evidence-based policy recommendation document

Participants will be provided with an electronic evidence-based policy recommendation document via email after random group allocation. This will have specific recommendations as to how the proportion of total allied health services should be delivered during weekends to align with current research evidence. Project investigators will develop draft recommendations through a consensus building approach based on the results of a systematic review and meta-analysis of the effectiveness and cost-effectiveness of in-patient weekend allied health services for improving patient and health service outcomes. In addition, a key stakeholder committee comprised of health professionals, managers, consumers, carer representatives, policy-makers, and academics will review draft recommendations and provide feedback before document finalisation. The document will be constructed in a simple format based on the Canadian Health Services Research Foundation [47]. This format allows for a one-page outline of key messages that have come from the research, a three-page executive summary, and 25-pages presenting the report findings and methodology.

#### Implementation strategy group 2: provision of an evidence-based policy recommendation document and access to a knowledge broker

Participants will be provided with the same electronic evidence-based policy recommendation document as implementation strategy group 1 via email after random group allocation. In addition, participants in implementation strategy group 2 will have access to a knowledge broker who will facilitate the transfer and exchange of relevant information between researchers and healthcare decision-makers to promote evidence-informed decision-making (EIDM) [34, 36]. A single knowledge broker with a Post-Honorary Doctorate (PhD) level qualification, from an allied health professional background, with research experience, currently employed as a post-doctoral research fellow will be recruited. The knowledge broker will offer a 60-min initial consultation with the allied health manager on a one-on-one basis via telephone, videoconference, or face to face (where able) to perform an individual, organisational, and external environment (e.g. government policy) needs assessment. Where required, the knowledge broker will assist development of a 12-month plan to address individual and organisational capacity for evidence-informed decision-making. One 60-min group-based webinar session will be offered within the first 6 months of the intervention period depending on allied health manager availability. Follow-up contact will be offered on a monthly basis via email or telephone, according to the manager’s preference, throughout the 12-month intervention period. Knowledge broker dosage (frequency, intensity, time, and type) was based on the description of a knowledge broker role implemented as part of a randomised controlled trial evaluating three knowledge translation strategies by Dobbins et al. [36]. The knowledge broker will follow an iterative process, with prompting questions informed by the COM-B (capacity, opportunity, motivation, and behaviour) model [48]. The number and format of contacts between the allied health manager and knowledge broker will record engagement with the knowledge broker implementation strategy.

### Outcomes

#### Primary outcome: alignment of weekend allied health service provision with policy recommendations at 12-month follow-up

Allied health service events (occasions of service) occurring during weekends, as a proportion of total allied health service events for the relevant hospital wards over a 1 month period will be used to determine alignment with the policy recommendation. This information will be collected at baseline for the preceding calendar month and the same corresponding calendar month at the 12-month follow-up. Allied health service events will be defined as per the 2017 National Allied Health Data Working Group (NAHDWG) endorsed National Allied Health Best Practice Data Sets (Additional file 1). Where data relating to allied health service events during weekends and weekdays is not available, data relating to allied health staffing levels or budgetary allocations during the weekend as a proportion of total allied health staffing levels or budgetary allocations for the relevant wards at each hospital for the preceding month will be used. Each participant cluster (hospital/health service) will receive a single classification as either (1) practice fully aligned with policy recommendation for both acute and sub-acute hospital wards, (2) practice partially aligned with policy recommendations (e.g. if practice on acute wards is completely aligned with the policy recommendation but not on sub-acute wards, and vice versa), or (3) practice not aligned with policy recommendation.

#### Secondary outcome 1: mean hospital length of stay at 12-month follow-up

The mean hospital length of stay for relevant wards over the 1 month period preceding random group allocation, and the same corresponding month 12 months later. Hospital length of stay is a key driver of hospital efficiency [49–52] and provides a measure of benefit or non-inferiority for weekend allied health provision [37, 38, 53].

#### Secondary outcome 2: opportunity cost to make the decision during the intervention period at 12-month follow-up in AUD$ (time to make decision, resources used, and knowledge broker time attributable to each participant in implementation strategy group 2)

Participants will record self-reported time (person-hours) taken to make the resource allocation decision, and any resources used (e.g. librarian to conduct literature search). For implementation strategy group 2, the self-reported data will be combined with time contributed by the knowledge broker to each decision-maker. The opportunity costs involved in making a decision (e.g. staff time and resources used) will be captured using interviews with allied health managers and logs of staff time recorded by research personnel. Measures of staff time will be valued using market salary rates in AUD$, with a 33% salary “on-cost” loading to account for allowances, leave, and other employee entitlements. Understanding the cost and benefits of providing research implementation strategies can assist healthcare governance agencies making implementation resource allocation decisions.

#### Process measures

Semi-structured interviews performed by a researcher who has not been involved in delivering the intervention at 12-month follow-up will be used to explore participant experiences concerning: (1) perceptions of the trustworthiness and sufficiency of evidence of the evidence base to guide clinical practice in this area of allied health service delivery, (2) the sources of information relied upon by allied health managers when deciding upon the model of weekend allied health service delivery they used in acute and sub-acute hospital wards and why they chose to use those sources, (3) perceived most influential source of information encountered by allied health managers when deciding upon the model of weekend allied health service delivery they used in acute and sub-acute hospital wards and why they thought this source was the most influential, and (4) perceived potential improvements to the intervention received and how it was provided. This information will be used to inform future allied health research implementation strategies.

#### Qualitative measure: perceived risks, barriers, and facilitators to adopting evidence-based policy recommendations at 12-month follow-up (implementation strategy groups 1 and 2) and during knowledge broker interactions (implementation strategy group 2 only)

Semi-structured interviews will explore what participants perceived as being the risks, barriers, and facilitators encountered in adopting or not adopting the policy recommendation. Control group participants will not be invited to participate in this final qualitative interview.

### Participant timeline

**Fig. 1:**
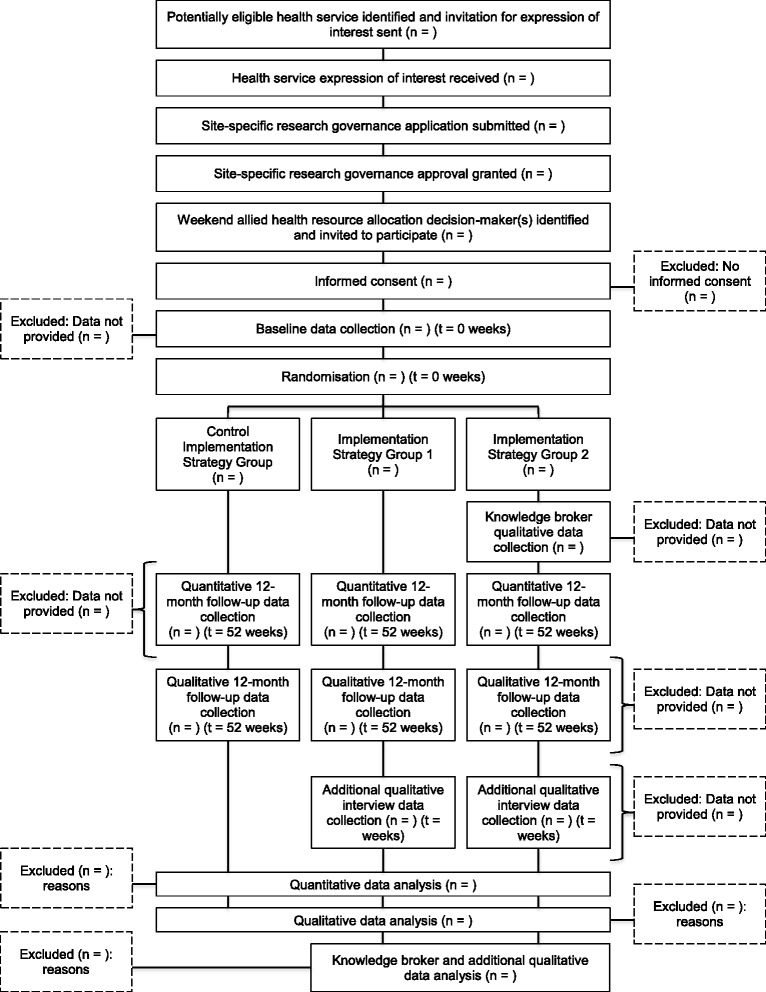
CONSORT study flow diagram

The Consolidated Standard of Reporting Trials (CONSORT) study flow diagram is provided in Fig. 1 [54]. Baseline data collection and implementation strategy group allocation are planned to occur following the participant information and consent process. A 12-month period will then be provided between initial implementation strategy provision and follow-up to allow sufficient time, on pragmatic grounds, to initiate changes required to align weekend allied health resource allocation with the evidence-based policy recommendation. Follow-up data will then be collected after the 12-month intervention period upon trial completion. The Standard Protocol Items: Recommendations for Interventional Trials (SPIRIT) flow diagram schedule of enrolment, interventions, and assessment procedures is described in Table 2 [55].

**Table 2:**
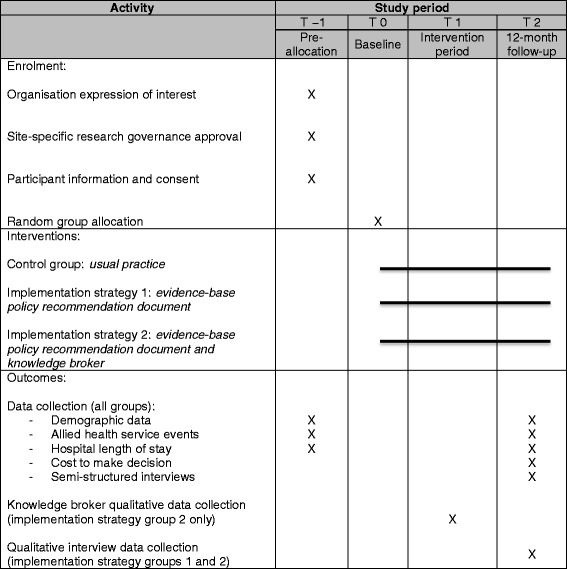
SPIRIT flow diagram: schedule of enrolment, interventions, and assessment procedures

### Sample size

The sample size estimate was calculated based on the units of assessment of the primary outcome ‘alignment of weekend allied health service provision with policy recommendations at 12-month follow-up’. The most conservative unit of assessment for this outcome will be clustered at individual hospitals/health services with geographically distinct decision makers. We therefore conducted our power analysis at this “cluster-level” rather than at the ward-level or individual decision-maker level (as one hospital/health service may have multiple decision makers involved). A sample size of 25 clusters (hospitals/health services) per group will provide greater than 80% power under the assumption that 50% of participants in an intervention group and 10% in the control group will completely align with the policy recommendation. We will aim to recruit 27 clusters (hospitals/health services) per group to allow for approximately 5–10% loss to follow-up in each group. Assumptions regarding statistical power and expected loss to follow-up were derived from a similar randomised controlled trial by Dobbins et al. [33]. Based on data from a survey of physiotherapy services provided outside business hours in Australian hospitals by Shaw et al. [41], and allied health staffing levels for health service inpatients in Victoria [56], it is anticipated that none of the recruited health services will be completely aligned with the evidence-based policy recommendation at baseline. This analysis was performed using Stata 13 (StataCorp, 2013. Stata Statistical Software: Release 13. College Station; TX: StataCorp LP).

### Recruitment

Project investigators will identify potentially eligible hospitals/health services using pre-existing professional networks and publicly available resources (e.g. government websites). Members of the research team will contact the allied health management either face-to-face, via telephone, or email and provide information regarding the study. The allied health management will then be asked if they support the research being conducted at their organisation. If support is provided, the potential participant/s responsible for weekend allied health resource allocation decisions at the health service will be identified via the allied health management. Potential participants (weekend allied health resource allocation decision-makers) will be contacted, provided with information on the study, and written informed consent will be sought for participation.

### Assignment and concealment of implementation strategy group allocation

Study investigators will consult with health service representatives to determine decision-making structure for weekend allied health services within their health network. Some health services in Australia are comprised of multiple geographically separated hospitals that report to the same board but have independent decision-making processes in relation to weekend allied health service provision decisions. They will be treated as separate units of recruitment and randomisation, where health service representatives report that decision-making for geographically distinct hospitals is independent and that they anticipate ability to prevent contamination of the intervention between sites within their network. Units of randomisation (hospitals or health services) will then be stratified according to geographic location. A random number sequence will be generated using an online software application (Sealed Envelope Ltd. 2018. Create a blocked randomisation list. [Online] Available from: https://www.sealedenvelope.com/simple-randomiser/v1/lists), incorporating permuted blocks of randomly selected sizes of three, six, or nine, and stratified according to metropolitan or regional status. This random number sequence will be generated and held by a single investigator (TPH) in a secure location so that investigators conducting recruitment and data collection are blinded to the allocation status of participating sites and the allocation of the next site to be recruited.

### Blinding

Participant weekend allied health decision-makers will not be blinded to group allocation. It will be clear to participants which implementation strategy group they have been allocated due to the nature of the trial, thus it is not possible to blind participants. In order to maintain allocation concealment, site allocation to implementation strategy group 2 will only be revealed to the knowledge broker once the site has been recruited and baseline data collected. Investigators performing data collection will be blinded to participant implementation strategy group allocation for the duration of the study. Investigators performing qualitative interviews will not be blinded. The trial data analyst will be blinded to group allocation. Three mock codes representing different sequence allocation will be used to blind the statistician conducting the final quantitative data analysis from the identity of each hospital/health service and the implementation strategy group allocation.

### Data collection

Project investigators will collect the primary outcome data by requesting an allied health activity statistics report for the relevant wards at each hospital from the weekend allied health decision-maker/s at each health service. It will be requested that this report contains the minimum variables: number of allied health service events, date of each allied health service event, as well as hospital and ward location of each allied health service event. Where these data are not available, allied health staffing levels or budgetary allocations during weekdays and weekends for the relevant wards at each hospital in the preceding month will be requested. The mean hospital length of stay for relevant wards will be collected as reported by the hospital electronic patient management systems. Previous research has shown this data collection method for hospital length of stay provides completeness of data capture when compared to other methods [49]. Allied health managers will be encouraged to record the amount of time (person-hours) taken and other resources used (e.g. librarian) to make the resource allocation decision in a log. For implementation strategy group 2, these data will be combined with knowledge broker records of the amount of time attributed to each participant during the intervention period. Project investigators will perform audio-recorded semi-structured interviews either face to face, via telephone, or videoconference as per participant preference. Qualitative data from knowledge broker conversations will be audio-recorded, and regular communication (e.g. email, phone, online forums) with the knowledge broker will be captured.

### Analysis

#### Quantitative

Primary outcome: alignment with policy recommendation will be analysed with pairwise comparisons (intervention group 1 vs control, intervention group 2 vs control, and intervention group 1 vs intervention group 2) performed using the sign rank test for ordinal data among matched pairs. This primary analysis will be conducted at both the cluster level and ward level. Analysis will be undertaken according to ‘as randomised’ (intention-to-treat) principles. Where it is identified that participants have moved between clusters allocated to different intervention groups during the study period, we will undertake a contamination-adjusted intention-to-treat analysis.

#### Qualitative

Semi-structured interview and data from knowledge broker interactions will be transcribed verbatim, with identifying data removed. An inductive thematic analysis process including constant comparison will used to analyse qualitative data [57]. Rigour in this qualitative study will be ensured by the strategies of immersion in data, reflexive analysis, memo writing, peer debriefing, and consensus coding between team members [58].

#### Economic evaluation

The economic analysis will calculate the “incremental cost per additional cluster and ward that completely align with the policy recommendation” of implementation strategy group 1 vs control, and implementation strategy group 2 vs control. The opportunity costs involved in making a decision, captured using interviews with decision-makers and research personnel logs of staff time involved, will be valued using market salary rates with a 33% on cost loading to account for leave and other employee entitlements. This analysis will be a trial-based evaluation. The analysis will then be fed into a net-benefit analysis which will incorporate data relating to the amount of allied health events captured at the baseline and 12-month follow-up assessments and changes in hospital length of stay. These data will then be modelled into a 5-year time horizon assuming that weekend service levels at the 12-month follow-up assessment are maintained 5 years into the future. One-way sensitivity analyses will be conducted to model the effects of having different numbers of allied health managers involved with making this decision at both the cluster and ward level.

### Monitoring study conduct

Monitoring of study progress will be performed at regular meetings between study investigators. Strategic governance oversight will review study recruitment progress, quality of data collection and management, and the occurrence of any unintended effects identified throughout study conduct. Adjustments shall be made to aspects of trial conduct as necessary; however, funding sources will not be involved in study monitoring or decisions regarding adjustment of trial conduct. Data collection at baseline and 12-months will provide an opportunity for monitoring of study progress. The knowledge broker shall also be able to provide feedback to the wider research team as to the study progress in implementation strategy group 2. There is no planned interim analysis, as only one study follow-up period has been planned.

### Ethics and dissemination

#### Research ethics approval

Approval to conduct this study has been obtained from the Monash Health Research Ethics Committee (HREC/17/MonH/44) and has been registered with the Australian New Zealand Clinical Trials Registry (ANZCTR) (ACTRN12618000029291). Universal Trial Number (UTN): U1111-1205-2621. Site specific research governance approval will be sought from each requesting health service upon review of the study protocol, participation information and consent form, and other requested documents (including subsequent modifications). Subsequent to initial review and approval, investigators will make safety and progress reports as requested.

#### Protocol amendments

Amendments to the study protocol will require approval from study investigators and the human research ethics committee. Any amendments will be communicated via trial registration updates, and reported in any published manuscripts associated with the study as necessary.

#### Consent

Potential participating allied health decision-makers will be provided with information regarding the study via the participant information and consent form. They will be provided with the opportunity to discuss the project with study investigators and time to consider their response. Return of a signed participant information and consent form will constitute informed consent for study participation. A copy of the signed participant information and consent form will be provided to the participant, and the researchers will retain a copy for their records. The Version 2, Master Participation Information and Consent Form (PICF) is presented in Additional file 2.

#### Confidentiality

The researchers will conduct themselves in accordance with the Declaration of Helsinki and the Principles of Ethical Conduct outlined in the National Statement on Ethical Conduct in Research Involving Humans [59]. All forms where the participant is identified (e.g. consent forms) will be kept in a locked filing cabinet in a lockable room, accessible by only the research team. Electronic data will be stored in password access folders on Monash University ‘LabArchives’. The details of data storage will be made available to participants who will not be identifiable in any literature published from the findings of this study.

#### Access to data

Information relating to the participation in the trial will not be available to any persons outside the study team. De-identified results data can be made available upon request to study investigators.

#### Ancillary and post-trial care

Usual practice control group participants will receive the evidence-based policy recommendation document upon trial conclusion.

#### Dissemination policy

A forum for allied health managers will be organised for the purpose of communicating the findings from this research. The results from the research will be reported in scientific journals and presented at conferences and workshops with personal information omitted. Participants will be advised they may request a copy of any results once available. De-identified data will be made available upon request to study investigators. Authorship eligibility will be determined according to the International Committee of Medical Journal Editors (ICMJE) recommendations.

## Discussion

Implementing research evidence into health policy and practice is actively promoted [23, 60]. The continuous process of disinvesting from low value healthcare practice and reinvesting in new approaches that are more efficacious, accurate, and safe, requires the integration of local expertise with the best available external evidence from systematic research [61]. Research in the allied health professions has identified the benefits of many healthcare interventions, such as strength and functional sensory discrimination training to reduce impairments for patients following stroke [62–66]. Innovations in allied health service delivery have also been made to improve access to, and reduce cost of interventions. Recent studies suggest that rehabilitation and exercise programs for chronic health conditions may be equally effective when delivered in home-based settings compared with centre-based settings, providing a potential alternative for those who cannot access centre-based programs [67–69]. The benefits of these research findings are clear. Yet, in order to produce desired outcomes at scale, evidence must be disseminated and implemented across healthcare organisations [70, 71].

Increased pressure on healthcare organisations to improve access, quality and cost of care has led to the identification of strategies to reduce the gap between research and practice [72]. As allied health professionals generally have positive attitudes towards evidence-informed decision-making [8–10, 17–19], strategies targeted at changing reactions, beliefs, and knowledge may not address the needs of decision-makers. Instead, the research implementation strategies described in our randomised controlled trial protocol aim to complement existing evidence-informed decision-making processes in allied health. Stroke rehabilitation is one of many areas where allied health has led the implementation of research into policy and practice [73]. Consensus implementation statements based on systematic review evidence [74] and clinical champions (diffusion fellows) [75] have been identified as some of the best methods for implementing stroke rehabilitation evidence into practice [73, 76]. While our strategies (evidence-based policy recommendation and knowledge brokerage) share similarities with consensus statements and clinical champions, these approaches have not yet been evaluated in a randomised controlled setting.

## Conclusion

Evidence-based policy recommendation documents communicate key research findings to the healthcare community in an accessible format. This comparatively low-cost research implementation approach can be combined with using a knowledge broker to work collaboratively with decision-makers to promote the transfer and exchange of information. The results from this study may also inform the development of a model for translating research into practice for allied health settings.

## Additional files

Additional file 1: Definition allied health service event. (DOCX 72 kb)

Additional file 2: Master Participant Information and Consent Form Version 2, 20/09/2017. (DOCX 38 kb)

## Abbreviations

ANZCTR: Australian New Zealand Clinical Trials Registry
COM-B: Capacity, opportunity, motivation, and behaviour
EBDM: Evidence-based decision-making
EviTAH: Evidence Translation in Allied Health
ICMJE: International Committee of Medical Journal Editors
NAHDWG: National Allied Health Data Working Group
PhD: Post-Honorary Doctorate
PICF: Participant Information and Consent Form
TIDieR: Template for Intervention Description and Replication
UTN: Universal Trial Number
