# Supplementary material for: Implementation of evidence-based weekend service recommendations for allied health managers: a cluster randomised controlled trial protocol
Source: Implement Sci. 2018 Apr 24;13:60. doi: 10.1186/s13012-018-0752-7 (PMC5916715; doi:10.1186/s13012-018-0752-7)
Supplement: Supplementary file 1 — Definition allied health service event. (DOCX 72 kb) [file 13012_2018_752_MOESM1_ESM.docx]

“A specific, time defined encounter during which a person (with a known patient identifier) receives a service from an allied health professional, assistant or student.

Each allied health service event occurs within an admitted patient episode of care, an episode of non-admitted patient allied health care or an emergency department stay.

Within each episode of care a person can have one or more allied health service events.

An Allied Health Service Event may span one or more days.

There may be one or more Allied Health Service Events provided by the same allied health professional, assistant or student on the same day.

An Allied Health Service Event must be individual patient attributable and can be direct and/or indirect. A person may or may not be in physical attendance for an Allied Health Service Event.

A person may Fail to attend an Allied Health Service Event.”

[2017 National Allied Health Data Working Group (NAHDWG) endorsed National Allied Health Best Practice Data Sets]
