# Supplementary material for: Implementation of evidence-based weekend service recommendations for allied health managers: a cluster randomised controlled trial protocol
Source: Implement Sci. 2018 Apr 24;13:60. doi: 10.1186/s13012-018-0752-7 (PMC5916715; doi:10.1186/s13012-018-0752-7)
Supplement: Supplementary file 2 — Master Participant Information and Consent Form Version 2, 20/09/2017. (DOCX 38 kb) [file 13012_2018_752_MOESM2_ESM.docx]

**Allied Health Evidence-Based Decision-Making Partnership Project**

**Participant Information and Consent Form (PICF)**

Chief Investigators

Professor Terry Haines

Professor Meg Morris

Professor Nicholas Taylor

Associate Professor Anne Holland

Professor Leeanne Carey

Dr Elizabeth Skinner

Dr Cylie Williams

Associate Professor Anne Bardoel

Professor Jenny Martin

Dr Lisa O’Brien

Principal Investigators

Dr Jennifer White

Mr Mitchell Sarkies

Ms Kathleen Philip

Ms Kellie Grant

Associate Investigators

Ms Wendy Hubbard

Dr Kelly-Ann Bowles

Professor Andrew Palmer

**1. Introduction**

The Allied Health Evidence-Based Decision-Making Partnership Project Committee invites you to participate in a nation-wide study assisting allied health managers to make evidence-based resource allocation decisions. This National Health and Medical Research Council (NHMRC) funded Partnership Project will recruit allied health managers from health services across Australia to a 12-month randomised controlled trial.

This Participant Information and Consent Form (PICF) tells you about the research project, explains the procedures involved and will help you decide if you wish to take part in the research. Please read this information carefully and feel free to ask questions about anything that you don’t understand or want to know more about. Participation in this research is entirely voluntary and your decision will not impact professional relationships with the research team.

If you decide you wish to take part in the research project, you will be asked to sign the consent section of this form. By signing consent you are stating that you:

- Understand what you have read;
- Consent to take part in the research project;
- Consent to take part in the research process as described;

You will be given a copy of this Participation Information and Consent Form to keep.

**2. What is the purpose of this project?**

The NHMRC funded Allied Health Evidence-Based Decision-Making Partnership Project Committee is developing an evidence-based policy recommendation document for allied health resource allocation during the weekends. We wish to test the effectiveness of providing this evidence-based policy recommendation or the provision of this same document with the addition of an expert knowledge broker to improve evidence-based resource allocation decision-making.

**3. What does participation in this research project involve?**

This is a national randomised controlled trial design

- If allocated to the control group, participants will experience a 12 month wait of usual care with no additional resources provided, until provision of an evidence-based policy recommendation document.
- If allocated to intervention group 1 you will be provided with the evidence-based policy recommendation document to help guide resource allocation decision making towards weekend allied health services.
- If allocated to intervention group 2, you will be provided with the same evidence-based policy recommendation plus access to an expert knowledge broker to provide extra help in making these decisions. The knowledge broker is a Post Doctoral Research Fellow from an allied health professional background who will help facilitate the transfer and exchange of relevant information to promote evidence-based decision-making. Regular communication (email, phone, online forums) with the knowledge broker will be available as required to sustain engagement and facilitate problem solving. Conversations discussing perceptions and experiences towards the policy implementation will be audio-recorded.

This project will be performed over a period of 12 months, with data collected at baseline and 12 month follow-up. If you wish to participate, you will be required to complete a survey interview for data collection at the beginning of the project and 12 months later at project conclusion. It is anticipated these survey interviews will take approximately 20 minutes to perform.

On completion of the study, allied health managers in each study group will be invited to participate in an audio-recorded qualitative interview exploring their experience of participation in this research and any barrier and facilitator to policy implementation.

Every participant (even those in the control group) will receive the evidence-based policy recommendation document upon trial conclusion.

**4. What are the possible benefits?**

If allocated to either of the implementation strategy groups you might benefit from provision of synthesised or “pre-processed” research evidence to inform weekend allied health resource allocation decision-making.

In addition, if allocated to implementation strategy group 2, the knowledge broker will provide a mechanism for explanation, assistance, and clarification to improve understanding of how research evidence applies to your organisation.

If allocated to the control group, you will still receive the evidence-based policy recommendation document upon project conclusion.

You may receive professional development benefits through seeking educational opportunities to improve evidence-based decision-making, and improve professional networks within and external to your health service, and with the academic sector. You may gain research interpretation skills and improved confidence in applying those skills to resource allocation decisions. This project provides an opportunity for quality improvement, and depending on decisions made, more efficient use of resources leading to improved patient outcomes and departmental budget management. The ultimate benefit will be the opportunity to develop skills in evidence-based decision-making and improve the resource allocation of allied health services during the weekend to practice that aligns with research evidence.

**5. What are the possible risks?**

This project is no different to usual practice where allied health managers make regular decisions around resource allocation. As such, we don’t believe this project presents any additional risks to participants beyond usual practice.

**6. Do I have to take part in this research project?**

Participation in any research project is voluntary and your decision will not impact professional relationships with the research team. If you do not wish to take part, you don’t have to. If you decide to take part and later change your mind, you are free to withdraw from the project and any stage, however you will no longer receive the research implementation strategy ongoing.

**7. How will I be informed of the results of this research project?**

Public presentations regarding this project will be made at conferences and the results will be published in a scientific journal. If you wish to receive a summary of project findings, please provide your contact details on the consent form.

**8. What will happen to information about me?**

The information we collect will be stored in a de-identified manner in a password-protected Monash University LabArchives file for a period of 7 years. Members of the investigative team will be able to access this information. Any information obtained in connection with this research project that can identify you will remain confidential and will only be used for the purpose of this research project. In any publication and/or presentation, information will be provided in such a way that you cannot be identified, except with your permission.

In accordance with relevant Australian and/or Victorian privacy and other relevant laws, you have the right to access the information collected and stored by the researchers about you. You also have the right to request that any information, with which you disagree, be corrected. Please contact one of the researchers named at the end of this document if you would like to access your information.

**9. Ethical guidelines**

Monash Health Human Research Ethics Committee (HREC/17/MonH/44) has approved the ethical aspects of this research project. Site specific Assessment has been approved by (insert site specific assessment).

This project will be carried out according to the National Statement on Ethical Conduct in Human Research (2007) produced by the National Health and Medical Research Council of Australia. This statement has been developed to protect the interests of people who agree to participate in human research studies.

**10. Who can I contact?**

If you want any further information concerning this project or if you have concerns about any aspect of your involvement in the project, you can contact the principal investigator:

Name: Jenni White

Telephone: +61 (0) 447 492 943

Email: [Jenni.White@monash.edu](mailto:Jenni.White@monash.edu)

**11. Complaints**

If you have any questions or concerns about your rights as a participant in this study, or if you have any complaints, you may contact:

Name: Deborah Dell

Position: Manager, Human Research Ethics Committees

Telephone: (03) 9594 4611

**12. Local Complaints**

If you have any complaints referring to the site specific conduct of this study, you may contact the local complaints person:

Name:

Position:

Telephone:

**13. Consent**

I have read this document and I understand the purposes, procedures and risks of this research project as described within it*.*

I have had an opportunity to ask questions and I am satisfied with the answers I have received.

I freely agree to participate in this research project as described.

I understand that I will be given a signed copy of this document to keep*.*

*Participant’s name (printed) ……………………………………………………*

*Signature Date*

*Declaration by researcher*: I have given a verbal explanation of the research project, its procedures and risks and I believe that the participant has understood that explanation.*

*Researcher’s name (printed) ……………………………………………………*

*Signature Date*

*Note: All parties signing the consent section must date their own signature.*

If you wish to receive a summary of project findings, please provide the best contact information for this to be sent to you below:

Email:__________________________________________________________________________________

OR

Postal address:________________________________________________________________________
